# Supplementary material for: Life history, nest longevity, sex ratio, and nest architecture of the fungus-growing ant Mycetosoritis hartmanni (Formicidae: Attina)
Source: PLoS One. 2023 Jul 26;18(7):e0289146. doi: 10.1371/journal.pone.0289146 (PMC10370743; doi:10.1371/journal.pone.0289146)
Supplement: S1 File — (PDF) [file pone.0289146.s002.pdf]

SUPPORTING INFORMATION, PLoS ONE 2023

Life history, nest longevity, sex ratio, and nest architecture of the fungus-growing ant *Mycetosoritis hartmanni* (Formicidae: Attina)

Ulrich G. Mueller[1], Anna G. Himler[1,2], Caroline E. Farrior[1]

[1] Department of Integrative Biology, University of Texas at Austin, Austin, TX 78712, USA
[2] Department of Biology, College of Idaho, Caldwell, ID 83605, USA

Page 2 Figure S1. Colony survivorship in a cohort of 150 established colonies of *Mycetosoritis hartmanni* over seven years between 2000-2007, estimated using four different modeling approaches.

Page 3 R-script used in the analyses of colony lifespan

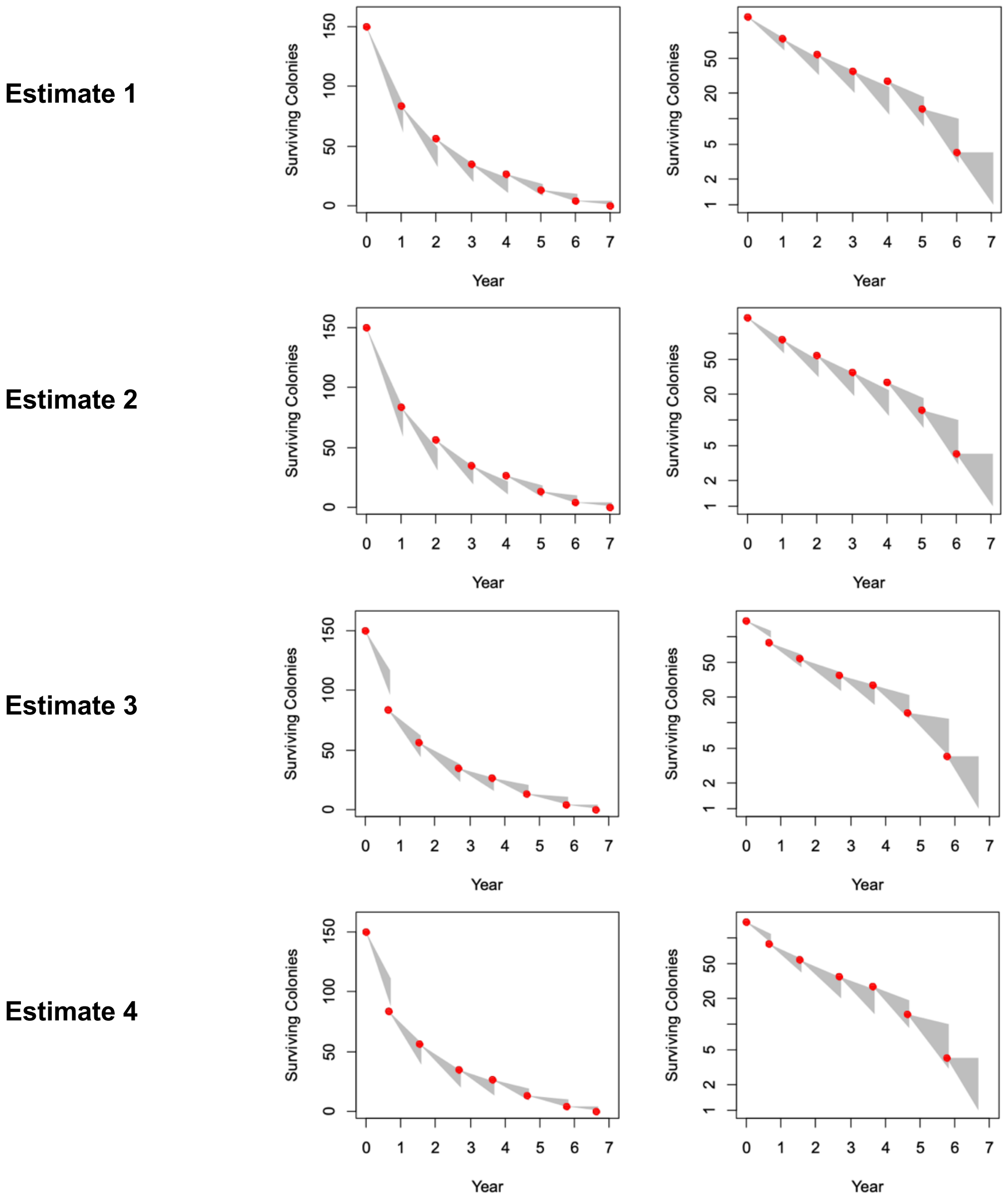

Fig S1. Colony survivorship in a cohort of 150 established colonies of *Mycetosoritis hartmanni* between 2000-2007 (Year 0 to Year 7), estimated using four different modeling approaches (Estimates 1-4) and plotted as the absolute number of surviving colonies over time (left graphs) and as the Log of the number of surviving colonies (right graphs). Because we did not always census the colonies on the same day of the year (the initial survey of the cohort of 150 colonies was in September, all later surveys of surviving colonies were conducted between April and July in subsequent years), and because mortality events may differ between seasons, we generated estimates making alternative assumptions covering the extreme possibilities: (i) mortality was assumed to occur only outside the re-survey season April-July (Estimates 1 and 3); or alternatively (ii) mortality risk was assumed to be constant across all days of the year including the re-survey months April-July (Estimates 2 and 4). Estimates 1 and 2 ignore differences in sample sizes across year (later years have naturally smaller sample sizes because of colony mortality), while Estimates 3 and 4 weigh confidence in the mortality rates across years by the number of individual colonies at the start of a given census interval. See also Table 1 in the main article and the modeling details in the R-script on page 3 of the Supporting Information.

R-script used in the analyses of colony lifespan

```
Ant colony mortality and lifespan. Best estimates.

#Data

nalive = c(150,84,56,35,27,13,4,0) #number of colonies observed alive by census

ctimes = c(0,238,560,980,1325,1690,2108,2423)  #census times in days since original observation
tf = length(nalive) #censuses
timeInts = ctimes[seq(2,tf)] - ctimes[seq(1,tf-1)] #days between each census

#Estimate of mortality if we average across years (bad estimate because there is much less data as time goes on)

#Estimate 1 - average over years, mort in winter

morts = (nalive[seq(1,tf-1)] - nalive[seq(2,tf)])/nalive[seq(1,tf-1)]
est1 = mean(morts)

#Estimate 2 - average over years, mort by day

morts = (nalive[seq(1,tf-1)] - nalive[seq(2,tf)])/nalive[seq(1,tf-1)]/timeInts*365.25
est2 = mean(morts)

survivalbyday=NULL
for(x in seq(2,tf)){
        survivalbyday = c(survivalbyday,(nalive[x]/nalive[x-1])^(1/timeInts[x-1]))
}
est2 = mean(1-survivalbyday^365.25)


#Estimate 3 - average by individual, mort in winter

samplesizes = nalive[seq(1,tf-1)]
morts = 1 - nalive[seq(2,tf)]/nalive[seq(1,tf-1)]
est3 = sum(morts*samplesizes)/sum(samplesizes)

#Estimae 4 - average by individual, mort by day

samplesizes = nalive[seq(1,tf-1)]
survivalbyday=NULL
for(x in seq(2,tf)){
        survivalbyday = c(survivalbyday,(nalive[x]/nalive[x-1])^(1/timeInts[x-1]))
}
morts = 1-survivalbyday^365.25
est4 = sum(morts*samplesizes)/sum(samplesizes)


#################
mu = c(est1,est2,est3,est4)

maximumlifespan = -1/mu*log(0.05)

averagelifespan = 1/mu
#################

for(est in mu){

        sbyday = (1-est)^(1/365.25)

        win.graph(width=8,height=4); par(mfrow=c(1,2))
        for(xx in seq(1,2)){
                if(xx==1) plot(ctimes/365.25,nalive,ylim=c(1,160),xlab="Year",ylab="Surviving Colonies")
                if(xx==2) plot(ctimes/365.25,nalive,ylim=c(1,160),log="y",xlab="Year",ylab="Surviving Colonies")

                for(i in seq(1,length(nalive)-1)){

                        x = rbinom(1e6,nalive[i],(sbyday)^timeInts[i])
                        qx = quantile(x,c(0.025,.975))

                        polygon(x=c(ctimes[i],ctimes[i+1]+20,ctimes[i+1]+20,ctimes[i])/
365.25,y=c(nalive[i],qx[2],qx[1],nalive[i]),col="gray",border=NA)
                }
                points(ctimes/365.25,nalive,col="red",pch=19,cex=1)
        }
}
```
