# Supplementary material for: Life history, nest longevity, sex ratio, and nest architecture of the fungus-growing ant Mycetosoritis hartmanni (Formicidae: Attina)
Source: PLoS One. 2023 Jul 26;18(7):e0289146. doi: 10.1371/journal.pone.0289146 (PMC10370743; doi:10.1371/journal.pone.0289146)
Supplement: Attachment — Submitted filename: Response Letter.pdf [file pone.0289146.s003.pdf]

Our Responses to Comments

Black: Comments by Editor, Reviewers, or Journal
Blue: Our responses

JOURNAL COMMENTS
3. We note that the grant information you provided in the 'Funding Information' and 'Financial Disclosure' sections do not match. When you resubmit, please ensure that you provide the correct grant numbers for the awards you received for your study in the 'Funding Information' section.
Our response: done

4. Thank you for stating in your Funding Statement: "The research was supported by funding from the National Science Foundation (Doctoral Dissertation Improvement Grant to AGH; CAREER award DEB-998379 and OPUS award DEB-1911443 to UGM); and the W.M. Wheeler Lost Pines Endowment from the University of Texas at Austin."
Please provide an amended statement that declares *all* the funding or sources of support (whether external or internal to your organization) received during this study, as detailed online in our guide for authors at http://journals.plos.org/plosone/s/submit-now. Please also include the statement "There was no additional external funding received for this study." in your updated Funding Statement.
Our response: We checked the listed funding information for accuracy (all information is correct and complete), and we added at the end of our Acknowledgements the sentence "There was no additional external funding received for this study."

Please include your amended Funding Statement within your cover letter. We will change the online submission form on your behalf.
Our response: We include the amended Funding Statement in our cover letter.

5. Thank you for stating the following in your Competing Interests section: "No competing interests."
Please complete your Competing Interests on the online submission form to state any Competing Interests. If you have no competing interests, please state ""The authors have declared that no competing interests exist.""
Our response: We changed our Competing Interest statement to "The authors have declared that no competing interests exist."

This information should be included in your cover letter; we will change the online submission form on your behalf.
Our response: We include the requested Competing Interest Statement in our cover letter.

6. Please include captions for your Supporting Information files at the end of your manuscript, and update any in-text citations to match accordingly.
Our response: Done; we added captions to label the two files in our Supporting Information, and we use in-text citations of "S1 Datasets" and "S2 Analyses" to refer to the two files in our Supporting Information:

S1 Datasets. Excel file summarizing raw data and metadata
S2 Analyses. Colony survivorship modeling and R-script

EDITOR COMMENTS
I agree with the reviewers that this is an interesting study that deserves publication, and I enjoyed the level of detail you provide. The reviewers suggested some edits that you might want to consider, and I'll add my own:

The introduction seems a bit redundant and/or unorganised. For example, you explain Wheeler's observations twice, once in the beginning and then later in more detail. The beginning of the introduction almost reads like an abstract, and some information in the introduction might fit better into the Methods section (e.g. references to the supplementary material).
Our response: We agree, many thanks. We have shortened our Introduction by eliminating information duplicated in different paragraphs, and we moved the sentence referring to our Supporting Information into the Methods.

The explanation of the species name also feels a bit out of place since it follows a paragraph that made me want to see the methods and the data – perhaps the species name discussion can come earlier in the introduction or later in the discussion?

Our response: We thought much about best placement of the two paragraphs on the history of the species name *Mycetosoritis hartmanni*. After trying out various places in our manuscript (none of which are perfect for these two paragraphs), we feel that the current placement between Introduction and Methods may be the best compromise. These two paragraphs are of greatest value for the taxonomist readers, who often include a historical note, or some side-remarks about etymology of taxon names, between the Introduction and Methods. Also, Reviewer 2 commented that she/he/they "appreciated the detailed explanation of the taxonomic history of this species and the account of Wheeler's early encounters with it", so we would like to keep the brief note on the species name *Mycetosoritis hartmanni* more visible right after the Introduction rather than bury it in the Methods or at the end of the Discussion.

REVIEWER #1: GENERAL OPINION:

The manuscript gives a nice (but in some cases incomplete, see below) overview about the biology of the poorly known fungus-farming Mycetosoritis hartmanni ant, mostly about its life history, longevity, nest architecture, phenology, sex ratio and survivorship. I'm basically happy to read such a detailed "naturalist" description about a species' biology and hope that this work will find its place in a high quality journal for publishing. Despite such knowledge on different species would be very important in the days of the climatic catastrophe, the "modern" stories are rather fashionables nowadays. I it should be the duty of the editors to consider whether such a story could be enough interesting for the PLOS ONE's readers. It will be, as I hope. In the case of refusing, I would encourage the authors to submit a rewritten version of the manuscript to some journal read basically by myrmecologists. The main research has been done about twenty years ago and I feel somewhere that the general description is not up-to-date. To tell the truth, despite I'm a myrmecologist who does not work with Attina ants, I clearly remember for some phylogenetic trees of this group. So, why the phylogenetic position of this ant is not discussed shortly in such a work which could be a review on the knowledges of a poorly known species?

Our response: We agree. We discuss in the second paragraph the phylogenetic position of *Mycetosoritis* among the so-called "lower Attina", and we summarize also prior discussions that address whether the two species *M. hartmanni* and *M. vinsoni* are separate species or may actually be one species. To provide more information, as requested by Reviewer 1, we added a sentence to the second paragraph to discuss the relationship of the genus *Mycetosoritis* (known only from North America) to the sister genus *Mycetarotes* (known only from South America), and we added citations to the relevant recent phylogenetic analyses (Sosa-Calvo et al. 2017, 2018; Hanisch et al. 2022).

On the other hand, there are some points that are mentioned in several places, see some examples below. It would be nice to read critically the full text by the authors and live the repeating thoughts only on the most relevant place.

Our response: We agree. We have removed duplicated information from the Introduction (see above, the Editor also recommended removal of some duplicated information in the Introduction), and we address all below comments in detail below.

MAJOR ISSUES:

-Line 1, §1, Page 3: could the authors give the type species of the genus?

Our response: Good idea, many thanks. We added to the information on the type species in parentheses after citing Wheeler (1907) "*hartmanni* is the type species of the genus *Mycetosoritis*".

-§2, Page 3 and/or some other part(s): I miss a more general overview about the known biology of this ant, in some short sentences. E.g. what about its colony founding, dietary, preferred climate and vegetation, and maybe about the topics discussed in this manuscript in detail? I know that such details are discussed in the manuscript sporadically but it would be better to see in the Introduction the summarised knowledge on this ant already known before this work.

Our response: Wheeler's (1907) species account did not report anything on "colony founding, dietary, preferred climate." We summarize in our review of Wheeler (1907) really all information reported by Wheeler at that time on the general biology of *M. hartmanni*; no one has subsequently studied this ant, and our study is the first since Wheeler (1907) to characterize anything about behavior, ecology, and biogeography of this ant species.

-Last §, P 3: It's nice that the nomenclature of this species is discussed. In this case the taxonomic name ("Mycetosoritis hartmanni (Wheeler, 1907)") and the original name ("Atta hartmanni Wheeler, 1907") also should be mentioned. Officially, the taxonomic name should be given in the 1st mentioning of the species in a work (currently at L6, P2).
Our response: We agree, and we added the following information to the first sentence of this paragraph, quoting the title of Wheeler's species & genus description: "*Atta (Mycetosoritis) hartmanni subgen. et sp. nov.*"

-Last sentence, §1, P4: "ICZN 1999. International Code of Zoological Nomenclature. Fourth Edition. The International Trust for Zoological Nomenclature, London, UK. 306 pp." (https://www.vliz.be/imisdocs/publications/271138.pdf) should be checked and cited.
Our response: We added a citation to the ICZN 1999 at the end of this paragraph.

-1st sentence of Materials and Methods: The authors basically worked on a single, about northernmost population of a relatively widely spread (from Louisiana-Texas to Honduras) species and later did some general conclusions on the biology of this ant. It would be important to emphasize in the Conclusion that the biology of other populations could be partly different, e.g. at the southernmost regions. See e.g. L10-13, §3, P14 for a population with different biology.
Our response: Good idea, many thanks. We have added a sentence at the end of the Conclusion referring to possible population differences: "Future research could address how the nest architecture, survivorship, and sex-ratio patterns reported here for a population in Central Texas compare to other populations across the range of *M. hartmanni* from the USA to Central America".

-About active or passive: The authors use active formula too much, as I feel (e.g. Last lines, P5: The authors start four sentences with "We" in a methodological part): I'm not a native English speaker and not sure how PLOS ONE prefers the frequent usage of active formula. However, I think the passive structure is better at drawing attention to the research, while the active structure is better at expressing the authors' own opinions.
Our response: We prefer to use the active voice, because we believe the active voice is more engaging for readers, and because our aim was to write a modern natural-history report in the engaging traditions of Thomas Belt, WM Wheeler, NA Weber, and EO Wilson.

-About citing: When there is a statement, its background should be clearly given which can be some citation and/or the results of the current work. E.g. the authors write withouth citation that "…mortality rates of small and incipient nests are typically high in ant species." (L9, §2, P7-8). Is it a thought? Or there are some minor cases, like (10-11, §2, P12): "Because July and August are the hottest and driest months in central Texas, with more regular rains resuming typically in late August or early September…"
Our response: We agree. We have added a reference to Hölldobler & Wilson 1990 to support the statement that "…mortality rates of small and incipient nests are typically high in ant species." We now cite the temperature-adaptation study in Texas by Mueller et al 2011 to support the statement that "Because July and August are the hottest and driest months in central Texas, with more regular rains resuming typically in late August or early September…"

-About the survival of the colonies (e.g. L last before, §2, P7): are the authors sure in that all the colonies have died of and not moved to some other places (e.g. because the changing of the vegetation, arriving of some aggressive ant colonies, etc.)? Please, discuss it.
Our response: We added a new paragraph to the *Results & Discussion* to discuss why colony migration is very unlikely:
"**Absence or rarity of colony migration**
The preceding section on colony survivorship presupposes that disappearance of a flagged nest in a specific location is due to colony mortality, not colony migration. This presupposition is justified for three reasons. First, we never observed any above-ground colony migration in *M. hartmanni* during our extensive observations of nest aggregations throughout the US range of this species. Second, it is difficult for fungus-growing ants to relocate nests above ground because they have to move their delicate fungal gardens in addition to any brood (pieces of fungus garden carried by workers dry and degrade quickly above ground). Both of these reasons suggest absence or rarity of above-ground colony migrations in *M. hartmanni*. Third, we never observed in our careful nest excavations any side tunnels that could enable any lateral below-ground migration (i.e., each of the 49 established nests that we excavated had only a single vertical tunnel linking garden chambers, never any side tunnels), suggesting absence or rarity of below-ground colony

migrations. Disappearance of nests in our long-term survey of a cohort of 150 established nests is therefore very unlikely due to colony migration, but due to colony death."

-About "queen may run out of stored sperm" (e.g. L7, §2, P8; L1, §1, P12 and in other parts): I feel it to be a bad speculation, especially in the case of a species which has so small colonies. Can the authors imagine that only few hundred-few thousands of sperms a queen receives during copulation? Do the authors know some similar stories at some other ant species? Could they cite them?
Our response: We agree, a queen is unlikely to run out of sperm if a queen mates successfully, but we would like to mention the possibility of rare sperm depletion, because we have observed in dissections of spermathecas of *Atta texana* that, whereas most queens have spermathecas filled to the brim with sperm, we have also observed some rare cases where a *texana* queen's spermatheca contained only a few sperm (possibly because the mating of the queen was interrupted during the intense male-male competition during mating flights). We have inserted the phrase "in rare cases" to this sentence to acknowledge that sperm depletion late in life is unlikely, so the sentence now reads "… or a queen may run out of stored sperm in rare cases and can no longer produce workers …".

-§1 of Results and Discussion: Could the authors give the total number of nests they examined?
Our response: The sample sizes are discussed in the first paragraph of the *Results & Discussion* (e.g., "a total of 39 established *M. hartmanni* nests" at Stengl LPBS …"), and sample sizes are also listed for each population in the first row of Table 2, which is cited multiple times throughout Methods and Results & Discussion. Table 2 will appear early in the Results & Discussion, so the information on sample sizes is available early to readers.

-It would be nice to see some photos about the ant and the nest in such a descriptive works. The authors cite some from iNaturalist (L5, §2, P9) but its stability is not guaranteed.
Our response: Good suggestion, thank you. We have added a new figure (new Fig 1) showing photos of the turriform mounds of *M. hartmanni*.

-About repetitions: e.g. it was not a new information for me, after reading the manuscript up to this part (PP15-18, §2, P9): "The steep entrance tumuli identifying M. hartmanni nests are easiest to find after a time of no rain during the second half of spring when colonies excavate most sand during nest expansion. This explains why we found during general ant collecting throughout Texas additional M. hartmanni populations frequently in spring, and less during other seasons." I had the similar feeling when I read (L11-12, §2, P10): "The largest nest had 148 workers, but worker number per nest is typically less than 100 workers in M. hartmanni." So, could the authors be more critical with repetition thoughts?
Our response: We have re-read our manuscript and reduced unnecessary repetition (e.g., see comment above regarding repetition in the Introduction). Where our findings replicate the observations by Wheeler 1907 (e.g., Wheeler's writing that nests seem to be easiest to find in spring, which we found also to be true), some repetition is unavoidable because our observations detailed in the Results & Discussion revisit Wheeler's observations that we summarize in the Introduction.

-End of §1, P10: Do we know something about the history and biogeography of the two discussed genera? So, maybe Mycetosoritis arrived from a tropical region and Mycetophylax from a continental one which may explain the difference about the wintering habit of these ants.
Our response: We perused the relevant recent biogeographic literature (e.g., the revision of the genus *Mycetophylax* by Sosa-Calvo et al. 2017; Branstetter et al 2017, who reconstruct ancestral dry/wet habitats, but not temperature habitats) and there exists too little information to justify any discussion in our manuscript on the biogeographic origins of the genus *Mycetosoritis* versus the genus *Mycetophylax*. Any discussion of adaptations to ancestral temperature habitats would be very speculative. Our guess is that both genera originated in warm tropical climates (*Mycetosoritis* likely in Central America; *Mycetophylax* presumably in tropical South America, the great majority of species in the revised genus *Mycetophylax* are tropical), but only a formal biogeographic character-state reconstruction involving representative *Mycetophylax* lineages and representative outgroups can really address geographic origins and ancestral adaptations. We decided therefore not to include any discussion on the ancestral adaptations to temperature by *Mycetosoritis* versus *Mycetophylax* lineages.

-About careful drafting: e.g. it is written (L1-2, §2, P10) that "…most colonies had between 20-70 workers, only two colonies had over 100 workers (118 and 148 workers)…". It would be right e.g. as: "…most colonies had between 20-70 COUNTED workers, only two colonies had over 100 COUNTED workers (118 and 148

workers)…". What about the foraging workers and the ones which escaped, etc.? Be more careful with such strong statements, please. Similarly, "…with nests of USUALLY 40-60 workers…" (L3, §1, P18) would be the right form because the authors mention e.g. 148 workers too.
Our response: We agree, we should phrase these results in terms of observed *counts*. We now write "… most colonies had counts between 20-70 workers, only two colonies had counts over 100 workers …". We also write in the Conclusion " … with nests of typically 40-60 workers …", as suggested by the Reviewer. We have changed our writing to "counts" also in several other sections throughout the manuscript.

-L6, §2, P10: Did these incipient colonies have brood?
Our response: We explain later in this same paragraph on "Semi-claustral nest founding" that "We found no large larvae or pupae in the incipient garden, but small brood may have been present hidden in the garden matrix." The other incipient nests in our study did not have a garden, and therefore also no brood.

-About "non-claustral nest founding" (e.g. P11): "SEMI-claustral COLONY founding" is better, as I know.
-L1-2, §2, P11: Why the lifespan of that queen has not been monitored during the years to really see the lifespan at least one queen?
Our response: We agree, good suggestion. We should have known better and written "semi-claustral" instead of "non-claustral." We changed all "non-claustral" to "semi-claustral."

-L4-6, §2, P12: Could the authors summarize the total developing time?
Our response: We are hesitant to give estimates for total developing time, even approximate range of developing time, it would be too much of a guess because we excavated nests at two-week intervals, and we did not follow development of individual brood in the lab. Moreover, our studies of development times in *Atta texana* have shown that length of development stages is very dependent on temperature, and also whether eggs are laid by foundress queens or older queens (Fang & Mueller 2019; Fang et al in prep). We believe our current narrative of when we found the first eggs, first larvae, first pupae, and first newly eclosed adults in field nests of *M. hartmanni* is sufficient to estimate approximate development times under the temperature conditions in the field.

-L16-17, §2, P12: "…suggesting that the foundress queen of the latter incipient nest had lost her garden…" Or has he not yet raised it?
Our response: We agree. We added that very recent nest initiation could be a second possibility. We now write: "… the foundress queen of this incipient nest had either very recently dug her nest and had not yet started her garden, or alternatively this foundress queen lost her garden because of drought, garden disease, or unknown factors."

-End of §1, P13: "This annual transition in spring from investment into colony growth and maintenance (investment into workers) versus into reproduction could be under queen control, but is more likely under worker control because workers would seem to have greater influence over the rearing conditions (e.g., nutrition, temperature) of developing female larvae." Could it be compared with some other ant species, or discussed in detail in some other way?
Our response: We are hesitant to add to this paragraph by elaborating on queen- versus worker-control in other ant species, but we added at the end of this sentence a reference to Pamilo & Crozier 1996, who discuss theory and empirical studies on parent-offspring conflict over sex ratio and colony growth in ants.

-L10-11, §2, P15: "…queens may therefore prefer the deeper chambers…" And/or have they retreated there at the disturbing?
Our response: Unlike other fungus-growing ant species (e.g., *Mycocepurus*), queens of *M. hartmanni* do not attempt to escape upon disturbance, but they hide in the nearest garden and curl up in a defensive position. This curling-up & hiding response by queens is the reason why we able to collect queens from almost all nests. We already discuss this defensive response of curling up in our manuscript.

-P16-17: Could the fungus be given on some taxonomic level? The Genbank accessions are good but it would be nice to know something about the taxonomy of the fungus during reading.
Our response: Thank you for pointing out this oversight. We now write: "The fungus cultivated by *M. hartmanni* in central Texas is an undescribed species in the genus *Leucocoprinus* (Genbank accession AF079727) that is sequence-identical …"

MINOR ISSUES:
-Line numberings would improve the discussion about the manuscript.
Our response: We sincerely apologize for the extra effort to comment on specific sections of our submitted manuscript. Failing to include line numbers was an oversight, and we appreciate the Reviewer's patience.

-Abstract: "(average 47.6 ± 31.9 SD, maximum 148 workers)" can be deleted, such details are not crucial for an abstract.
Our response: We deleted the estimate of standard deviation, but we prefer to give some specifics in the Abstract because some readers will be interested in some details on colony size (like average and maximum colony size), and we therefore write now in parentheses "(average 47.6 workers, maximum 148 workers)".

-L7, §2, P2 and in several other cases: The sentence starts with abbreviated genus name. I'm not sure whether it's a preferred format by the journal, many other journal do not like this style.
Our response: Correct, our mistake, we need to spell out the genus name if appearing at the beginning of a sentence.

-L11, §2, P2: dots are missing after "M" in two cases
Our response: Thank you, we corrected these two typos.

-L2 vs. L4, §2, P7: Maybe I do not understand it well but how 150 nests can be "additional" which already have been "pre-flagged"?
Our response: We deleted the "additional", we agree this is confusing.

-L1-2, §1, P8: Repetitions: "…differences generated small differences in our mortality estimates. Estimates…"
Our response: We agree. We now write "… sample-size differences generated small discrepancies in our estimates of mortality. Estimates 1 and 2 ignore …".

REVIEWER #2:
The authors present a straightforward and engaging natural history of the seldom encountered fungus-farming ant, *Mycetosoritis hartmanni*. This study represents the only quantitative account of seasonal behavior, nest architecture, reproduction, and colony longevity in this species. I appreciated the detailed explanation of the taxonomic history of this species and the account of Wheeler's early encounters with it. Especially interesting was the authors' observation of changes in sexual investment across the breeding season and the estimate of colony lifespan. The conclusions are well-reasoned and follow the data well. A few minor changes and additions would improve this manuscript and increase its utility for other biologists.

My main critique: You repeatedly mention that colonies selected for your study could be operationally defined as "mature" because they were large and appeared to be more than one year old. For most ant species, there is a minimum number of workers (threshold) necessary for reproduction (which defines sexual maturity). Because you excavated and censused colonies with sexual present in some seasons, I think you likely have information about the minimum number of workers necessary for colony reproduction/maturity – but you have not reported this. It is my strong preference that the term 'mature' only be used if sexual were observed or if the minimum worker number (proxy) was observed outside of the mating season. If you don't want to define the colonies by their actual size/worker number, I would call them something else: perhaps "large", "active" or "visible." We know from studies of at least 3 Pogonomymex species that some colonies may stay small for many years, and never reproduce (probably because of resource limitation or genetic incompatibilities with mates). Size, age, and maturity should not be conflated.
Our response: We agree, thank you for pointing out the important conceptual distinctions between colony size, colony age, and colony maturity. We follow the reviewer's suggestion to eliminate the term "mature", and we use now the more conservative "large colonies" or "larger colonies" (per suggestion by the reviewer) to refer to those colonies that we selected in the field for our excavation and survivorship studies. In cases where we discuss colonies that have grown past the high-mortality stage typical for young colonies, we now use the term "established colonies" (older than 1 year) to differentiate from "young or small colonies" (less than 1 years old, or small mound). We define "established colonies" in both the Methods and in the Results & Discussion sections.

We also thank Reviewer 2 for the suggestion to include an estimate of the "minimum number of workers necessary for colony reproduction". In the section with subheading *Brood production, time of mating flights, and incipient nests*, we include now the following short paragraph addressing our best estimate for minimum colony size required for alate production:
"The average count of workers in the 15 nests where we found alate reproductives was 41.8 (± 20.6 SD), and of these 15 nests, the four nests with fewest worker counts had between 17-24 workers (Excel Sheet 5, S1 Datasets). Approximately 20 workers may therefore be the colony size minimally required for alate production, but this estimate of 20 workers is likely an underestimate of the true colony-size threshold for alate production because some workers escaped during our nest excavations and some foraging workers may have been absent from nests at the time of our excavations."

Materials and Methods:
1. "The focal population consisted of several hundred M. hartmanni nests distributed across two adjacent clearings in semi-open pine-oak forest." Please list the dominant pine and oak species present, as well as the more abundant, co-occurring ant species. Doing so will greatly increase the utility of this manuscript for future biologists seeking populations of M. hartmanni.
Our response: Good idea, thank you. We added the following sentence to describe species composition of the forest: "The two dominant tree species in this forest were loblolly pine (*Pinus taeda*) and post oak (*Quercus stellata*), followed by juniper (*Juniperus virginiana*) and occasional blackjack oak (*Quercus marilandica*), with yaupon (*Ilex vomitoria*) dominant in the understory."

2. "because wet weights are better estimates of resource investment by ant colonies into reproductives than their dry weights." I disagree with the above statement. Many studies have found important seasonal and age-specific investment strategies by analyzing the proportion of lipids (extracted with organic solvents) + lean weight (dry, fat free), which provide more nuance than wet weight alone - especially among ants that divide labor between interior and exterior roles in arid environments. You might rephrase to say " Wet weight is one accepted way of measuring resource investment by ant colonies and can act as a proxy for….".
Our response: We agree, and we now use the wording suggested by the Reviewer: "… because wet weight is one accepted proxy of resource investment by ant colonies into reproductives (Crozier and Pamilo 1996)."

3. "Any queen senescence and sperm shortage in M. hartmanni unlikely affects colony survivorship already at our estimated average…."
Typo: change to "……is unlikely to affect colony survivorship…"
Our response: done, thank you for spotting the typo.

Fire ants are especially efficient with sperm use (just 3 sperm cells per fertilization event). However, queens do run out of sperm around 6 years of age – suggesting that sperm limitation may affect colony longevity for other ant species as well (see work of Tschinkel). We don't know how much sperm your focal species stores, because you did not dissect the spermatheca. Therefore, I am not sure your statement is justified.
Our response: Our argument here is that sperm shortage is unlikely to impact colony survivorship at the average colony lifespan of 1.9-2.5 years, which leaves open the possibility that sperm shortage may occur at more advanced ages near maximum lifespan. We expanded this sentence to refer to this possibility: "… but sperm depletion could potentially be a problem at ages close to the maximum lifespan".

Results/Discussion:
4. Did you excavate colonies before foragers departed each day? If not, you might mention that colony sizes are estimates that may be conservative because of absent foragers.
Our response: We agree, and we expanded this sentence to refer now to this possibility: "… because some workers likely escaped during nest excavation, particularly early in the 2000 season when we had less experience excavating nests, and because some foraging workers may have been absent from a nest at the time of excavation."

5. Regarding colony reproduction: Is there a single, coordinated mating flight or multiple mating flights per season? If there were multiple mating flights, were sexuals collected between events or before the first flight? I may have missed your description of the mating flights.
Our response: We have never observed a mating flight of *M. hartmanni*, and we assume that there are several mating flights throughout the season because we found alates in nests from July – September (we already discuss the possibility of several mating flights in our manuscript). We added the following sentence to speculate on the time of day of mating flights: "We never observed a mating flight of *M. hartmanni* during

the mornings and early afternoons when we were present at our main study site to excavate nests, so mating flights may occur at dawn, during the evening, or possibly at night."

6. What was the ratio of sexuals to workers in your colonies (Mean and standard deviation)? Did larger colonies produce more sexuals? Did colony size influence sex ratio? You have lots of untapped data here.
Our response: Great suggestions. We tested for statistical significance of the correlations suggested by Reviewer 1:

We added to the section on *Brood production*: "The number of workers found in each of the 15 nests with reproductives was statistically significantly positively correlated with the respective number of total alate brood found in each nest (Pearson correlation coefficient r = 0.562, p = 0.0291), but not statistically significantly correlated with the respective number of total alate adults found in each nest (Pearson correlation coefficient r = 0.4152, p = 0.1238) (Excel Sheet 5, S1 Datasets). Because alate reproductive depart regularly from nests during mating flights, it is perhaps no surprise that worker number is not correlated to the number of alates found in each nest, even though we found evidence that larger nests with more workers generate more alate reproductive."

We added to the section on *Sex ratio*: "The number of workers found in each of the 15 nests with reproductive was not statistically significantly correlated with respective numerical sex ratios (Pearson correlation coefficient r = 0.049, p = 0.862) or with investment sex ratios (Pearson correlation coefficient r = 0.096, p = 0.733) (Excel Sheet 5, S1 Datasets). Given the protogynous sex-ratio transition of *M. hartmanni* during the season (Fig 3), these findings are perhaps no surprise, because nests with few and with many workers were excavated early and also late in the season, obscuring any effects that colony size may have on sex ratio."

7. "The size of the third chamber likely depended on the age of a nest, with younger nests having smaller third chambers because the workers added a third chamber only recently to a nest and this third chamber had yet to be enlarged by the workers."
Are you certain that all of these colonies were younger, and not just smaller?
Our response: We agree, and we now list both colony age and colony size as factors potentially explaining the size of a third chamber: "… size of the third chamber likely depended on the colony age or size, with younger or smaller nests having smaller third chambers …"

8. Your description of the garden was a joy to read. It is beautifully described. I was left longing for a photograph. Consider adding an image of an excavated garden and the ants.
Our response: We decided not to include images of garden or garden-tending workers in our manuscript here, and we reserve such images for an in-prep illustrated book of the fungus-growing ants of the USA.

9. "It is possible that M. hartmanni may therefore relocate gardens seasonally between different depths to optimize temperatures and moisture for garden growth and brood development, as occurs also in the sympatric leafcutter ant Atta texana (Mueller et al. 2011) and some subtropical leafcutter ants of South America (Lapointe et al. 1998; Bollazzi et al. 2008)." Jon Seal also showed annual, vertical movement of fungus gardens in Trachymyrmex septentrionalis. (Seal and Tschinkel 2006)
Our response: We added the Seal&Tschinkel reference and now write: "… as occurs also in the sympatric leafcutter ant *Atta texana* (Mueller et al. 2011), the higher-Attina *Trachymyrmex septentrionalis* in the south-eastern USA (Seal and Tschinkel 2006), and in some subtropical leafcutter ..."

10. "On 12. May 2000, we noticed that several of our flagged M. hartmanni nests, located in a more shaded area at the margin of our main study site at Stengl LPBS, had been overrun by an encroaching nest of the fire ant Solenopsis invicta. Such instances appear to be infrequent, however, perhaps because fire ants avoid sun-exposed dry areas, like the two forest clearings at our study site where M. hartmanni nests were most abundant." Another relevant citation here may be Josh King's PNAS paper about how fire ants do not displace native ants and do not survive/invade in undisturbed habitat.
Our response: We added the King & Tschinkel 2008 reference to the second sentence.

11. Fig 2: "Average sex ratios were calculated by first calculating the sex ratio for each individual nest, then averaging sex ratios across all the nests collected within a 2-week time period (data in columns O & P in Excel Sheet 1 in Supporting Information)." Consider reporting variance for the averages.
Our response: Variances are listed in the Excel file in the Supporting Information.
