# Supplementary material for: Life history, nest longevity, sex ratio, and nest architecture of the fungus-growing ant Mycetosoritis hartmanni (Formicidae: Attina)
Source: PLoS One. 2023 Jul 26;18(7):e0289146. doi: 10.1371/journal.pone.0289146 (PMC10370743; doi:10.1371/journal.pone.0289146)
Supplement: Attachment — Submitted filename: Response to Reviewers.pdf [file pone.0289146.s004.pdf]

Our Responses to Comments

Black: Comments by Editor & Reviewers
Blue: Our response

EDITOR COMMENTS
I enjoyed the manuscript and ruled this a minor revision to give you the opportunity to consider the last open comments - this might be difficult in the proof stage.

REVIEWER COMMENTS
1. If the authors have adequately addressed your comments raised in a previous round of review and you feel that this manuscript is now acceptable for publication
Reviewer #1: All comments have been addressed
Reviewer #2: All comments have been addressed

6. Review Comments to the Author
Reviewer #1: Thank you very much for considering my suggestions. I accept all of your answers and have only two minor requests. After managing these, I think this MS does not need some further review procedure:

1) Lines 64-65: The taxonomic names are not in a right form. The describer and year should be separated by a coma. Furthermore, when a species was described in another genus than the recently valid one, the describer and year should be closed to parentheses. So, the valid names are (as I gave in my comment): “M. hartmanni (Wheeler, 1907)” and “M. vinsoni Mackay, 1998”. See, e.g., antweb.org.

2) Lines 306-312 vs. 532-543: there are many repetitions. I think, it would be enough to write down these speculative thoughts only once and write a “see below/above” or “see Colony survivorship/Absence or rarity of colony migration” at the deleted part.

Reviewer #2: (No Response)

Our response: We have addressed the two comments by Reviewer 1:
(a) We now use in the third paragraph of the Introduction the taxonomically correct forms of the species “*M. hartmanni* (Wheeler, 1907)” and “*M. vinsoni* Mackay, 1998”, per recommendation by Reviewer 1.

(b) We edited lines 306-312 vs. 532-543 to reduce the appearance of repetitions. We added a cross-reference “as already noted by Wheeler (1907) (see above)” to the section in lines 306-312; and we deleted from lines 532-543 the subclause “that most nests accumulate in spring when workers expand nests after inactivity in winter” because that same information is already presented in the Introduction. We are hesitant to delete additional information, and we feel the writing in these two sections is appropriate and helpful to the reader.

We correct in lines 342 and 388 two minor typos that we overlooked in our previous revision (these corrections are highlighted in the PDF recording in TrackChanges all our edits since the last revision).

We also double-checked all the references; each citation appearing in the main text is listed among the References, and there is no redundant publication listed in the References that is not cited in the main text.
